# Influences on visit retention in clinical trials: Insights from qualitative research during the VOICE trial in Johannesburg, South Africa

**DOI:** 10.1186/1472-6874-14-88

**Published:** 2014-07-28

**Authors:** Busisiwe Magazi, Jonathan Stadler, Sinead Delany-Moretlwe, Elizabeth Montgomery, Florence Mathebula, Miriam Hartmann, Ariane van der Straten

**Affiliations:** 1 Wits Reproductive Health and HIV Institute, Faculty of Health Sciences, University of the Witwatersrand, PO Box 18512, Johannesburg, South Africa; 2 Women’s Global Health Imperative, RTI, San Francisco, CA, USA; 3 Center for AIDS Prevention Studies, Department of Medicine, University of California San Francisco, San Francisco, CA, USA

**Keywords:** HIV prevention, PrEP, Microbicides, Visit retention, South Africa

## Abstract

**Background:**

Although significant progress has been made in clinical trials of women-controlled methods of HIV prevention such as microbicides and Pre-exposure Prophylaxis (PrEP), low adherence to experimental study products remains a major obstacle to being able to establish their efficacy in preventing HIV infection. One factor that influences adherence is the ability of trial participants to attend regular clinic visits at which trial products are dispensed, adherence counseling is administered, and participant safety is monitored. We conducted a qualitative study of the social contextual factors that influenced adherence in the VOICE (MTN-003) trial in Johannesburg, South Africa, focusing on study participation in general, and study visits in particular.

**Methods:**

The research used qualitative methodologies, including in-depth interviews (IDI), serial ethnographic interviews (EI), and focus group discussions (FGD) among a random sub-sample of 102 female trial participants, 18 to 40 years of age. A socio-ecological framework that explored those factors that shaped trial participation and adherence to study products, guided the analysis. Key codes were developed to standardize subsequent coding and a node search was used to identify texts relating to obstacles to visit adherence. Our analysis includes coded transcripts from seven FGD (N = 40), 41 IDI, and 64 serial EI (N = 21 women).

**Results:**

Women’s kinship, social, and economic roles shaped their ability to participate in the clinical trial. Although participants expressed strong commitments to attend study visits, clinic visit schedules and lengthy waiting times interfered with their multiple obligations as care givers, wage earners, housekeepers, and students.

**Conclusions:**

The research findings highlight the importance of the social context in shaping participation in HIV prevention trials, beyond focusing solely on individual characteristics. This points to the need to focus interventions to improve visit attendance by promoting a culture of active and engaged participation.

## Background

Adherence has emerged as a particularly critical issue for clinical trials of user-dependent technologies such as Pre Exposure Prophylaxis (PrEP), delivered as tablets or vaginal gels [1-3]. Understanding what shapes adherence to investigational products has therefore generated much attention in the recent literature [4]. There is broad agreement that adherence is an individual behavior but is shaped by a complex mix of social, cultural, and economic factors [5]. The demographic and behavioral characteristics of trial participants, the acceptability of investigational products, and the community context, may all have a bearing on adherence decisions and capabilities. Regular supply of investigational product however is a fundamental precondition for trial participants to be adherent, and this requires regular attendance at clinic visits. In a systematic review of the barriers and facilitators to adherence in 24 HIV prevention trials conducted in African countries, low adherence in three studies was due to the lack of study product when participants missed clinic visits, and eight trials reported absenteeism from the study area as a major barrier to adherence, implying lack of access to product due to missed visits [6]. Moreover, at clinic visits, trial staff do not only dispense product and administer adherence counseling, but also monitor participant safety and assess trial endpoints.

Studies of visit retention in HIV prevention trials have focused on socio-demographic factors such as age, mobility, and risk behaviors [7,8]. Of particular concern in PrEP and microbicide trials is the focus on younger women, who are most at risk of HIV acquisition [9,10]. Their vulnerability to infection is attributed to the ‘complex interplay of biological, social, cultural and political factors’ [11], that underlie powerlessness to negotiate ‘safer sex’ , and insist on condom use [12]. These same social characteristics contribute towards instability and poor prospects for being retained in trials than older, more stable individuals. Younger women tend to have high rates of discontinuation or being lost to follow up [7]. Highly mobile populations are also difficult to retain due to the frequency and duration of study visits [13].

Visit retention may be supported by various strategies: participant-tracking; home and workplace visits; short messaging service (SMS) reminders; global positioning system (GPS) locators; improving the quality of clinical services; rewards and incentives for visit completion [13]. These strategies are primarily individual motivational strategies. Yet, trial participation can be influenced by the social and cultural contexts and everyday life processes. Relationships within the domestic domain with family members, partners, and within communities may constrain intentions and decisions to participate in a trial. While reasons for missing clinic visits may often be idiosyncratic and related to individual characteristics, there is a need to better understand the social context in which trials take place and its influence on participants’ abilities to keep to a clinic visit schedule.

The recently completed VOICE trial (MTN-003) was a Phase 2 B safety and effectiveness study of daily Tenofovir 1% gel, Tenofovir Disoproxil Fumarate tablet and Emtricitabine/Tenofovir Disoproxil Fumarate tablet for the prevention of HIV infection in women. The trial enrolled a total of 5029 HIV negative women in three countries (Uganda, Zimbabwe and South Africa) [14]. As a planned ancillary qualitative study to VOICE, VOICE-C was conducted at one site, Johannesburg, South Africa, concurrently to the VOICE trial. This sub-study aimed to explore contextual barriers and facilitators to women’s use of the investigational product and their experiences in the VOICE trial. We used data from the VOICE-C study to explore the type of influences on clinic attendance in the VOICE trial, and implications for future HIV prevention trials.

## Methods

The Johannesburg site for the VOICE trial was located in Hillbrow, an inner city neighborhood. The trial recruited participants from within Johannesburg, and as far as Soweto and Orange Farm townships located about 40 kilometers from the trial site. Participants in the VOICE trial were expected to complete monthly visits for HIV testing, safety assessment and product re-supply over a period of 12 to 36 months, depending on the date of their enrolment. Clinic visits were scheduled to occur every 28 days following enrolment, with an allowable visit window of 14 days before and 13 days after this target date. At the product use end visit (PUEV), participants were followed for a further eight weeks before finally exiting the study. The VOICE protocol defined a missed clinic visit as non-attendance within the visit window. Data on missed visits was recorded on a missed visit form.

### VOICE-C study sample

A random sub-sample of 165 VOICE participants was preselected for enrolment into VOICE-C. The eligibility criteria included: enrolment in VOICE; use of the investigational products for at least three months; not pregnant; HIV negative; and not on extended study product hold. Participants who were selected to participate in VOICE-C, signed a separate informed consent agreement, and their demographic details were recorded on a standard case record form (CRF). Further details of this sample are published elsewhere [15].

### Qualitative data collection

Participants were randomly assigned to one of three interview modalities: a once-off in-depth interview (IDI); serial ethnographic interviews (EI) repeated up to four times; or a focus group discussion (FGD). IDI and FGD took place in a private interview room in the VOICE study clinic, while EIs took place at the participant’s residence, or a venue of their choice. The serial nature of the EIs, combined with their greater focus on the participant’s everyday life and surroundings, provided researchers with an opportunity to become better acquainted with interviewees, learn about changes to their life experience over time, and in some cases to observe their living circumstances. Two trained female interviewers (BM and FM) conducted interviews in a language preferred by the participant (IsiZulu, SeSotho, Setswana, Xitsonga, and English). All interviews were audio-recorded, transcribed, and translated into English, and reviewed to assess accuracy. VOICE-C participants received fifty Rand [ZAR 50 = approx. 5 USD] as a reimbursement for transport and time.

Participant anonymity was maintained by using participant identification numbers (PID) on all study material. Personal information (names and places) mentioned in interviews and focus group discussions was censored in the transcripts and replaced with pseudonyms.

### Data analysis

The study team members coded translated transcripts in Nvivo (QSR International version 9.0). A socio-ecological framework that explored those factors that shaped trial participation and adherence to study products, guided the analysis. A codebook with 14 key codes was developed to standardize subsequent coding and an acceptable level of inter-coder reliability (ICR) was set at more than 80%. At least two team members coded approximately 10% of the transcripts in order to monitor ICR. Our analysis includes coded transcripts from seven FGD (N = 40), 41 IDI, and 64 serial EI (N = 21 women). Demographic and visit attendance data were tabulated using SAS (version 9.3, Cary, NC). Comparison between the VOICE and VOICE-C samples at the Wits RHI site used baseline demographic data collected at the VOICE enrolment visit.

### Ethical review

The VOICE-C protocol was reviewed and approved by the Human Research Ethics Committee of the University of the Witwatersrand (Ref: 090906) and the Institutional Review Board at RTI International, overseen by the regulatory infrastructure of the NIH, and monitored by FHI360 and RTI International.

Our research adheres to the standards contained in the Qualitative Research Review Guidelines (RATS) (http://www.biomedcentral.com/authors/rats).

## Results

Between July 2010, and August 2012, 144 randomly selected participants were screened for VOICE-C eligibility during or following their VOICE month three study visit. Of the 144, 19 were already lost to follow up from the parent trial and not screened, two declined screening, and four were not eligible for inclusion. Of the remaining 106 that consented to participate and were subsequently enrolled in VOICE-C, 41 completed an IDI, 40 participated in one of seven exit FGD, 21 completed two to four serial EIs, and four participants did not complete any of their planned interviews, resulting in a total of 102 participants.

We identified the following key themes as barriers or facilitators to clinic visits: social relations with intimate partners, household members, extended kin, friends and co-workers; employment and school attendance; clinic procedures and clinical care.

### Participant characteristics

Overall, 80% of VOICE-C participants (n = 102) were aged 18 to 30 years, 68% had completed secondary school, and 57% were employed either formally or informally (Table 1). At the time of their first interview, only two participants were no longer in a sexual relationship, 22% were married and 87% provided financial support and/or cared for their own children or that of their relatives. Although all respondents lived in Johannesburg, more than two thirds (71%) identified their natal home as being outside the Johannesburg Metropolitan area, in other provinces, or outside the borders of South Africa. There were no significant differences in demographic characteristics between participants included in VOICE-C and those not included in the VOICE-C cohort (N = 252) [15].

**Table 1:** Demographic characteristics of VOICE-C participants

**Variables**	**N**	**Percent**
Age group		
18 -- 30	80	78
31 -- 40	22	22
Education		
Secondary incomplete	33	32
Secondary complete	49	48
Attended college/university	20	20
Partnerships		
No sexual partner	2	2
Has a sexual partner	100	98
Income		
Earns income	58	57
No income	44	43
Child support		
Supports children	89	87
Does not support children	13	13
Natal home		
In Johannesburg	30	29
Outside Johannesburg	72	71
Totals	102	100

### Clinic visit attendance

VOICE participants had a mean of 10.4 follow-up visits (range: 0–21). 44% of VOICE-C participants missed at least one visit whereas 62% of non-VOICE-C participants missed at least one visit (ChiSquare test, p = 0.002). The mean number of visits missed was lower in the VOICE-C participants, compared to those not included in VOICE-C (1.55 vs. 3.79, Wilcoxon Test, p = 0.0003), and more VOICE-C participants were retained at the PUEV visit compared to their non-VOICE-C counterparts (97% vs. 87%; ChiSquare test, p = 0.003).

The following were identified through qualitative interviews as influencing visit attendance during the VOICE trial, viz. family, partner and household demands; work place, school or community expectations; and the study clinic itself. These are presented in more detail below.

### Mothers, daughters, partners – social obligations to others

Participant clinic attendance was influenced by social expectations from intimate partners, household members and extended kin. Overall, VOICE-C participants expressed concerns about disclosing their trial participation within these relationships, as they were uncertain about how those close to them would respond to this information. For example, a participant disclosed to her husband, but not to her sister in-law about her participation:

Hey, I think they will say why am I doing this and think I am a bit immoral, and such things. I don’t know what they will think. At times I don’t feel comfortable discussing my private life (age 24, IDI).

Failure to disclose participation in the trial influenced visit attendance. A participant suggested that one of the reasons why other participants missed their clinic visits could be because,

Most of them are not able to use the products and also [could not] come to the study [clinic] because they did not tell people in [their] homes that they are coming to the study [clinic] (age 38, IDI).

Women anticipated that their intimate partners would react negatively to their participation in the trial. For example, one woman (age 36, IDI) described her partner as ‘a bully’ and feared he would prevent her from visiting the clinic; she decided to conceal her trial participation. A 20-year-old woman’s partner objected at first because he felt that the time commitment for clinic visits would interfere with her responsibilities as a wife and income earner. However, women who initially experienced resistance from their partners often obtained approval after they explained the study and convinced their partners of the health benefits provided by the trial, including HIV testing. Despite their reservations, some male partners also supported women’s intentions to participate in the trial by giving them transport money and reminding them of clinic appointments. For instance when the outreach team could not contact a participant on her phone after she missed her visit they contacted her partner (she had given consent for the clinic to contact her partner) and he promised to bring her to the clinic:

He fetched me from my house…he asked me why didn’t I go because they [study staff] said I missed my visit. I said my phone is not working. So he brought me here [to the trial clinic] (age 19, FGD).

Similarly, the attitudes of parents, siblings and other household members towards trial participation influenced visit attendance. A 23-year-old participant concealed her participation in the trial from her parents, and when her mother visited her and found out about her participation she insisted that her daughter withdraw from the trial. However, she continued with the trial and attended visits when her mother was not present. On the other hand, household member support also had a positive impact on clinic attendance, especially for younger women still living with their parents. One young woman recalled that her mother told her: ‘Go and do what is good for your life…I don’t have a reason to stop you’ (age 20, IDI). A 26-year-old participant said that although her mother did not remind her about clinic visits, she supported her indirectly by looking after her small child when she visited the clinic.

Family and kinship obligations within a wider social network were felt strongly by VOICE-C participants. For many (71%) their natal home was located in another province or country, far from Johannesburg. Participants regularly travelled home to visit extended kin on religious holidays and to participate in rituals like funerals and weddings that re-establish kinship bonds. A 35-year-old woman missed four visits to attend her husband’s funeral in Zimbabwe and to observe the mourning period and cleansing rituals. Another younger participant said that her father ‘forced her’ to visit home regularly, resulting in frequent missed VOICE visits (age 21, EI). However, when participants were able to notify the trial of prolonged absences from Johannesburg in advance, women were given extra study products to cover missed visits.

### In search of a better life – work-seekers and students

The majority of participants had financial obligations to support family members. More than half (57%) of VOICE-C participants were employed as servers in restaurants and hotels, cashiers and packers in supermarkets, security guards, cleaners, administrators, and receptionists. In many of these situations, the hours were long and presented difficulties for attending regular monthly clinic visits. Even those who worked night shifts were too tired or had other domestic responsibilities to attend to during the day. One said, ‘The thing is I nearly dropped out because I could see that it [the trial] was putting me under pressure’ (age 25, IDI). Being employed did not necessarily imply stability, as many opportunities were for short-term contract work. The constant pursuit of employment contributed toward the itinerant nature of many trial participants, as this woman described:

In this area people move around a lot; today I am staying here, next month or in two or three months I am no longer here I am in another area. I am not working and I might get a job any time soon (age 31, IDI).

Moreover, employers were generally unsympathetic to requests for time off from work, even when presented with a clinic attendance letter from the trial clinic. A woman employed as a cleaner elaborated: ‘when I told her [the employer] that I have to go to the clinic, she just didn’t understand [agree]. So, I had to dodge [skip work] sometimes when she is not there’ (age 20, IDI). Personal circumstances also changed throughout the duration of the trial. An unmarried participant found a job as a nurse, and missed five consecutive clinic visits because of her demanding working hours and infrequent time off (age 23, FGD). Trial participants who attended school and university had similar experiences. A college student described college life as ‘hectic’; she missed two visits and ran out of tablets (age 20, IDI).

Participants were apprehensive about disclosing their participation in the trial to employers and workplace colleagues because of the public secrecy that still pervades HIV and AIDS. A participant noted that her work colleagues would gossip about her and say: ‘uh, uh you are lying maybe you are sick, why are you taking tablets every day? Maybe you have HIV/AIDS’ (age 31, IDI).

### The clinic context

Participants’ narratives of their experiences within the clinic setting reflected both negative and positive influences of the clinic environment on their participation, and clinic attendance. On the negative side, women often referred to the lengthy time spent waiting in the clinic. Study procedures for the monthly visit were approximately one and half-hours long, while quarterly visits (‘long visits’) could take up to two and a half hours. However, waiting time was dependent on the number of participants at the clinic. Participants expressed their frustration with waiting time: ‘You wait for the whole day to wait [to be seen] for 30 minutes, just imagine’ (age 30, EI). ‘Now if I am going for a long visit, I will start feeling like today I am going to be ill by the end of the day’ (age 30, EI). Lengthy clinic visits were extremely disruptive and demotivating, as this woman stated:

Yes, it is the issue of time. Sometimes you would find that as a person you have other plans, and they get spoilt because you have to come here to the study and find [do] their long visits. When you have to attend your visits then all your plans have to stop, and that is really irritating, hey. I once missed my visit and I ended up telling myself that I won’t be part of the study anymore because I missed my visit (age 25, EI).

Dissatisfaction with the time spent in the clinic could result in feeling discouraged to return: ‘once you think Oh my God I will sit on those couches [in the waiting area] for a long time, that is what makes one to be lazy to come here again’ (age 19, IDI). A participant observed another participant who had been waiting for some time: ‘she was waiting and not being attended to. She decided to leave saying she will never tolerate such a thing, she just left like that’ (age 21, EI).

Waiting time was particularly inconvenient for working women. ‘I did not mind waiting at the clinic before, but now I am working and I must be on time’ (age 21, EI). Another indicated that her time was worth money: ‘If I lose four hours at work that means I’m losing four hundred and fifty Rand [ZAR 450 = approx. 45 USD] for that day’ (age 28, FGD). The long time spent waiting in the clinic was not only an inconvenience, but was perceived as a lack of consideration for participants’ time. Yet, unemployed women felt this way too, as they played multiple roles as child minders and housekeepers, and were often extremely busy. ‘I dislike coming here [to the clinic] because we wait too long. In the future do not be surprised when I do not come here if there is no one at home’ (age 23, IDI).

For a few participants, the absence of public transport or living far from the clinic created difficulties to visit the clinic. Participants who resided far from the clinic and reached home after dark raised additional concerns about personal safety. However, participants could notify the clinic and arrange for a pickup and drop off service to a central point from which they could catch public taxis and busses.

While waiting time was a disincentive to attend clinic visits, the quality of care and positive encounters with trial staff promoted participation, especially in comparison to experiences of low standards of care in public health facilities and the high cost of private health care services. The VOICE participants valued the clinical services that they received for free such as pap smears and hepatitis B vaccination. They commented on the friendly and respectful manner in which the nurses interacted with them in comparison to government clinics. ‘Even though the waiting time was long, the VOICE nursing sisters were very respectful’ (29, FGD). In cases when participants missed their visits, the efforts to retain them in the study were regarded very positively. This participant missed her visits due to work commitments but returned for her next clinic visit after receiving a phone call from the clinic.

Hmm, what I like about the VOICE staff members is that they are more patient than any other studies that I have ever participated in. Like, at the beginning of the year I was busy at work and I missed my clinic visit. I thought maybe they have withdrawn me from the study. I was so surprised to receive a call from the VOICE staff member who said ‘you can come tomorrow and after the clinic visit we will give you a lift to work. The nurses will also give you the clinic attendance letter.’ The VOICE staff members encouraged me to continue with the study because before they phoned me I thought that I have messed up everything and I do not qualify to be in the study anymore. They are very supportive (age 30, EI).

Regular HIV testing also positively motivated women: ‘it makes me feel that I have to maintain my [HIV negative] status. I want to take care of myself by making sure that every month when I visit VOICE I am fine’ (age 21, EI); ‘So, when they test you every month and you discover that you are still negative you get excited’ (age 25, EI). The positive and supportive attitudes of the nurses encouraged women to share their personal problems: ‘friendly nurses whom I would speak to like they are my friends. Somehow that made me feel special’ (age 19, IDI).

Reimbursements were also an incentive to visit the clinic, as this was not only used for transport, but for personal and household items. ‘I used it to pay my accounts and so on. Yes it was very important because who is going to buy clothes for my siblings’ (age 24, IDI). Employed participants also appreciated the reimbursement. A single mother of one child who works as a waitress said: ‘when I am broke in the middle of the month I am able to buy some foods and other things’ (age 32, IDI).

## Discussion

Although overall trial retention in VOICE was satisfactory, participants did not attend monthly clinic visits consistently, thereby disrupting study product resupply and counseling, and missing safety assessments. Inconsistent clinic visits may therefore have contributed toward the overall poor adherence rates reported for the VOICE trial [14]. Seeking to understand the reasons for varying visit attendance, we found that women performed multiple social roles that competed with their participation in the trial. We also found that the trial design and expectations may not take into account the social context in which they are undertaken, and may need to be adapted to suit the exigencies of participants’ everyday lives.

Discourses about retention in clinical care and research often use the label ‘defaulter’ , implying that participants intentionally miss study visits. There are a number of complex reasons why trial participants miss visits [16]. Likewise, our findings suggest that missed visits are frequently unintentional, and due to factors outside individual participant’s locus of control. Participants were members of kinship networks, wives, girlfriends, caregivers, income earners, and scholars, and fulfilling these roles often compromised their ability to attend clinic visits. Clinic visits interfered with household responsibilities, work and school. As such, trial participants encountered difficulties when trying to balance their commitment to these different domains. Employers and educational authorities were usually inflexible, and constrained trial participants’ ability to attend clinic visits. On the other hand, kinship relationships also proved to facilitate participation in the trial; male partners and fellow household members were often supportive of participants and assisted them in attending their clinic visits. In this regard, disclosure about trial participation was central to whether support was offered or not. In addition, despite their negative experiences of waiting for long periods of time in the clinic, women liked the benefits of the trial, and acted as responsible participants by re-scheduling visit dates, and requesting extra supplies of product.

Our findings point to a problematic aspect inherent in the protocols and procedures that govern the running of clinical trials. Trial protocols often operate under the assumption that participants are ‘autonomous agents’ and are relatively free to participate. They seldom address the dynamics of gender, age, and social relations that may impact on the running of the trial [17]. These issues may be managed if incorporated into trial design. For example: mobile services could ensure consistent access to product even to those who do not manage to come to the clinic; visits could be made less frequent and at times that are suitable for those employed and in school, reducing the burden on participants; waiting time could be reduced and time spent in the clinic could be made more meaningful by providing recreational and learning opportunities.

Our research highlighted the importance of the quality of care provided by clinicians, the relationships established with the clinic staff as well as the flexibility in accommodating participants’ lives [18]. There is a need for innovative approaches to promote greater awareness of the importance of clinical trial participation that could engender support from household members, male partners, employers, educators, and the community [19]. Clinical trialists can also learn from the relative successes in retention in care and treatment settings. For example, the establishment of patient support clubs has been effective in retaining patients in antiretroviral (ARV) treatment settings [20]. Similarly trial participant groups or clubs may provide participants in prevention trials with a form of social support and opportunities for information sharing. This may also foster the creation of a new identity of being a study participant. Moreover, capitalizing on the gratitude expressed by participants for the provision of quality health services, and creating a culture of ‘active participation’ and engagement may increase participants’ sense of being valued and their commitment to the trial [21].

We recognize that this study has some limitations. Participants in this sub-sample had higher retention rates than their non-participating trial counterparts, and there may be other reasons for missed visits about which we are not aware. While the VOICE trial was conducted in 15 sites in Uganda, Zimbabwe, and South Africa, VOICE-C was undertaken at only one of these sites located in the inner city of Johannesburg; our findings may therefore not be generalized to other VOICE sites, or to other clinical trials. The strength of our findings, however, lies in the use of multiple qualitative methods that provided insights into the social context of women’s daily lives, and the influence that this has on trial participation.

## Conclusions

As we continue the search for HIV prevention options for women living in sub-Saharan Africa, we need to recognize that the women who are most at risk of HIV and who participate in our trials are young, upwardly mobile and have ambitions beyond that of being passive participants in a clinical trial. Young women have competing ambitions that make participation in a clinical trial challenging. By creatively changing the setting or the culture of the clinical trial itself, researchers may be able to make it more attractive and engaging.

## Abbreviations

ARV: Antiretroviral; CRF: Case record form; EI: Ethnographic interview; FGD: Focus group discussions; HIV: Human immunodeficiency virus; ICR: Inter-coder reliability; IDI: In-depth interview; MTN: Microbicide Trial Network; NIH: National Institutes of Health; NIAID: National Institute of Allergy and Infectious Diseases; NICHD: National Institute of Child Health and Human Development; NIMH: National Institute of Mental Health; OCTAVE: Office of AIDS Research-supported Online Collaborative Training for AIDS Vaccine Evaluation; PrEP: Pre-exposure prophylaxis; PUEV: Product use end visit; SAS: Statistical analysis system (Software); UKaid: United Kingdom Aid; USD: United States Dollar; VOICE: Vaginal and oral interventions to curb the epidemic; Wits RHI: Wits Reproductive Health and HIV Institute; ZAR: South African Rand.

## Competing interests

The authors declare that they have no competing interests.

## Authors’ contributions

BM: Collected data, analyzed data and contributed to drafting the manuscript. JS: Developed the concept, analyzed data, and contributed to drafting the manuscript. SDM: Developed the concept and contributed to drafting the manuscript. EM: Analyzed data and commented on the manuscript. FM: Collected data, analyzed data, commented on manuscript. MH: Analyzed data and commented on the manuscript. AVS: Analyzed data and commented on the manuscript. All authors read and approved the final manuscript.

## Pre-publication history

The pre-publication history for this paper can be accessed here:

http://www.biomedcentral.com/1472-6874/14/88/prepub
